# Some Disabilities from War Injuries

**Published:** 1916-07-08

**Authors:** 


### The Hospital

The Workers' Newspaper of Administrative Medicine and Institutional Life, Administration, National Insurance and Health.

No. 1570, Vol. LX. SATURDAY, JULY 8, 1916. PRICE ONE PENNY.

### SOME DISABILITIES FROM WAR INJURIES.

What disabilities—economic, social, political—the war will inflict on the nation as an organised unit time alone can tell. Even those who believe there are redeeming influences in the struggle recognise that such good as results is inevitably mingled with much that is evil, and that evidences of the latter will not readily disappear. To take one of the most mechanical influences, the free and irresponsible spending of money is an experience which without doubt carries its penalty, and there are ills more severe and more penetrating than this one. It may be hoped that our masters and pastors are alive to the responsibility which the present state of matters creates, and that what remedies are available are being used wisely and energetically. A sign not unwelcome is the organisation of some kind of effort to promote not merely economy, but the actual saving and investment of money. By co-operation a generous rivalry is encouraged, and what the individual may fail to do when dwelling in isolation he may accomplish with ease when associated with others set on a common quest. As we are often here concerned with hospital economies and with the application of organisation in the collection of funds, we may be allowed a sympathetic word for attempts to encourage these principles in the promotion of sound personal finance. It is those who adopt such principles who will be able to face with equanimity the reaction which cannot fail to follow the enormous national expenditure demanded by the existing situation. Personally we doubt the extreme prophecies of the pessimists, but that there must be a time of financial stress is beyond challenge; and preparation for peace is a measure of prudence to which the wise will give heed.

Here, however, we are concerned rather with disabilities which are bodily and mechanical, and which fall upon the individual in such a fashion as to make him of lessened value as a worker and social unit and perhaps directly or indirectly a charge on the community. Some of these are terrible enough in all conscience and it is a matter of national responsibility and duty that all of them be met, so far as they can be met, by help both sympathetic and practical. When so many appeals claim attention it must never be forgotten that our own men—men who have fought and bled for us—have the first claim. It is not so much a matter of charity as of duty and gratitude, and there are instances not a few in which adequate repayment or recognition or reward is quite beyond attainment. Whether such positions are to be met by public or private funds need not be argued for the moment. Everyone is agreed that they must be met by one or the other, and it would not be surprising to find that a combination of the two operations was better than either of them apart. Certainly for some of the worst ills the public conscience would not be at rest unless with any help given there is the association of human tenderness and the delicacy of that sympathy which is expressed, though words are not its channel. There may be mentioned here the pathetic position of young men smitten down in the fullness of life and condemned for the rest of their days to utter helplessness; what may be done by gentleness and skill must be done, and an endeavour to secure this at the new Home at Richmond is being pressed. Such an appeal must touch all our sympathies, and everyone should be proud to take some share. Similarly there are the men who have lost their eyesight, and though to speak of compensation for such loss would be a mockery, very substantial help is here possible. By education and training the blind man, it is well known, may become sufficiently expert not merely to earn a livelihood but to gain keenness, interest, and happiness for his own life.

Again funds are needed and also the personal help which cannot be bought but which carries the newly-blinded man through the terrible period when his loss falls upon him in its full stress. Such help by kindly hands and voices is being provided, but all may co-operate through interest and sympathy. The same things in substance are true of men who have lost one or more limbs, or who have been disfigured in consequence of injuries to the head and face, and the chapter can be unfortunately extended in many different directions. The point which we wish to make here is one often emphasised in these columns—namely, that in a much enlarged degree there is an opportunity for personal service and personal sacrifice on behalf of the sick and suffering, and that the general claim is now reinforced by considerations of justice, of patriotism, and of gratitude.

There are other disabilities suffered by our fighting men which, if less pathetic than many already mentioned, are of great economic importance alike to the individual and to the community. They deserve attention, first, because those who suffer them have done so in defending the nation's cause, and secondly, because, given proper treatment, many of the victims can be restored to full value as working units. Two groups may be mentioned in this classification: the one, injuries which have produced stiffness of joints and muscular wasting; the other, functional nervous disturbances which may for convenience be named neurasthenic. That much is being done in both of these directions we know full well, and we are the more inclined to recognise such work seeing that it brings to the doctors engaged in it little in the shape of fame or reward. But it is certain that in these two fields there is very much more to be done if the suffering men are to be saved from drifting into personal and civic uselessness. Naturally the more severe cases attract attention first, and for these there is a fairly large body of organised help. But equally the slighter cases need attention and they by no means demand less judgment and skill in treatment. The restoration of a man to the work of the nation is a considerable achievement, and this especially when the strength and youth of the community are being lavishly spent in war.

